# Leveraging Methylation Alterations to Discover Potential Causal Genes Associated With the Survival Risk of Cervical Cancer in TCGA Through a Two-Stage Inference Approach

**DOI:** 10.3389/fgene.2021.667877

**Published:** 2021-06-02

**Authors:** Jinhui Zhang, Haojie Lu, Shuo Zhang, Ting Wang, Huashuo Zhao, Fengjun Guan, Ping Zeng

**Affiliations:** ^1^Department of Epidemiology and Biostatistics, School of Public Health, Xuzhou Medical University, Xuzhou, China; ^2^Center for Medical Statistics and Data Analysis, School of Public Health, Xuzhou Medical University, Xuzhou, China; ^3^Department of Pediatrics, Affiliated Hospital of Xuzhou Medical University, Xuzhou, China

**Keywords:** Cox linear mixed-effects model, prediction model, gene expression, DNA methylation, two-stage inference, potential causal gene, cervical cancer, aggregated Cauchy association test

## Abstract

**Background:**

Multiple genes were previously identified to be associated with cervical cancer; however, the genetic architecture of cervical cancer remains unknown and many potential causal genes are yet to be discovered.

**Methods:**

To explore potential causal genes related to cervical cancer, a two-stage causal inference approach was proposed within the framework of Mendelian randomization, where the gene expression was treated as exposure, with methylations located within the promoter regions of genes serving as instrumental variables. Five prediction models were first utilized to characterize the relationship between the expression and methylations for each gene; then, the methylation-regulated gene expression (MReX) was obtained and the association was evaluated via Cox mixed-effect model based on MReX. We further implemented the aggregated Cauchy association test (ACAT) combination to take advantage of respective strengths of these prediction models while accounting for dependency among the *p-*values.

**Results:**

A total of 14 potential causal genes were discovered to be associated with the survival risk of cervical cancer in TCGA when the five prediction models were separately employed. The total number of potential causal genes was brought to 23 when conducting ACAT. Some of the newly discovered genes may be novel (e.g., *YJEFN3*, *SPATA5L1*, *IMMP1L*, *C5orf55*, *PPIP5K2*, *ZNF330*, *CRYZL1*, *PPM1A*, *ESCO2*, *ZNF605*, *ZNF225*, *ZNF266*, *FICD*, and *OSTC*). Functional analyses showed that these genes were enriched in tumor-associated pathways. Additionally, four genes (i.e., *COL6A1*, *SYDE1*, *ESCO2*, and *GIPC1*) were differentially expressed between tumor and normal tissues.

**Conclusion:**

Our study discovered promising candidate genes that were causally associated with the survival risk of cervical cancer and thus provided new insights into the genetic etiology of cervical cancer.

## Introduction

Cervical cancer is one of the most common malignancies in the female population, mostly caused by infection with human papillomavirus (HPV) (Šarenac and Mikov, 2019). In terms of cancer statistics in 2018, cervical cancer is the fourth most common malignancy and the fourth leading cause of cancer death among women worldwide, with an estimate of 570,000 cases and 311,000 deaths globally (Bray et al., 2018). Moreover, cervical cancer is the second primary cause of cancer death in women aged 20–39 years (Siegel et al., 2019). Although great advances have been achieved for cervical cancer, there is still a lack of reliable diagnostic biomarkers for early identification and screening (Chen and Gyllensten, 2015). In addition, despite the utilization of HPV vaccines for prevention and chemoradiotherapy as well as radical surgery offering satisfactory survival rate for early-stage cervical cancer patients, effective treatments for advanced patients are rarely available, especially in developing countries and regions (Chen and Gyllensten, 2015).

Therefore, it is an urgent demand in clinical practice that valuable biomarkers should be well discerned and validated to be a sign of the early stage or to provide profile of cervical cancer progression (Nahand et al., 2019). As an effort to understand the genetic foundation of susceptibility to cervical cancer, in the past decade multiple genome-wide association studies (GWASs) were undertaken and discovered a group of cervical-cancer-associated genetic variants (see Chen and Gyllensten, 2015), where a large number of associated germline genetic variants and genes were described for cervical cancer. These findings imply that the development of cervical cancer relies to a significant extent on inherited genetic components and genetic predisposing factors may affect the probability and persistence of, or sensitivity to, HPV infection and the rate of tumor development as well as progression (Chen and Gyllensten, 2015). However, like many complex human traits and diseases (Visscher et al., 2017), the genome-wide SNP-based heritability of cervical cancer estimated in GWAS is smaller than expected. For example, the heritability is 11.7% (*se* = 9.8%) in a Japanese population (Masuda et al., 2020) and 24.0% (*se* = 2.9%) in a Swedish population (Chen et al., 2015), both of which are lower than that observed in family studies (Magnusson et al., 2000). The remaining missing heritability suggests that a large number of causal genes and genetic variants have yet been discovered and that continuous efforts to identify causative genes for cervical cancer are worthwhile (Chen and Gyllensten, 2015).

As also demonstrated in many studies (Zhao et al., 2006, 2014; Wang et al., 2013; Zhu et al., 2017; Kim et al., 2018; Yu et al., 2020), mRNA-gene expression measured at the transcript level influences the progression of complex diseases more directly than other omic measurements. However, the establishment of the potential causal relationship between altered gene expressions and the survival of cervical cancer patients is not straightforward in observation studies due to unknown confounders and possible reverse causation. The latter is of particular concern because we cannot determine whether the regulated gene expressions are the causal factors or the consequence of the development or progression of cervical cancer due to the considerably complicated biological network and interaction. Due to this reason, previous studies often aimed to examine association rather than causality between gene expression and cervical cancer.

In statistical genetics, a powerful statistical tool to determine causal relationship and estimate causal effect of the exposure on the outcome in observational studies is Mendelian randomization (MR), which is built based on commonly used instrumental variable approaches developed in the field of causal inference (Angrist et al., 1996; Greenland, 2000; Sheehan et al., 2008). Under some certain assumptions, the results of MR analysis are less susceptible to reverse causation and confounding factors (Davey Smith and Ebrahim, 2003). A key point in MR is to select valid instrumental variables for the exposure (i.e., expression level). Biologically, methylation CpG sites of a specific gene within the transcript start site can downregulate its expression level, which can in turn further affect the survival of cancer patients (Glinsky, 2006; de Tayrac et al., 2009; Fabiani et al., 2010; Wang et al., 2013), indicating that methylation alterations play a central role in cancers by regulating expression profile. This motivates us to propose a two-stage causal inference approach with methylations as instrumental variables of expression to detect potential causal genes for the survival of cervical cancer patients. The two-stage instrumental variable inference is widely employed in many research fields, such as sociology, economics (Angrist and Keueger, 1991), and genetic medicine (Xue et al., 2020). In addition, applying methylations as instruments for causal inference is also commonly seen in recent genomic integrative analyses (Hannon et al., 2018; Qi et al., 2018; Wu et al., 2018).

**FIGURE 1:**
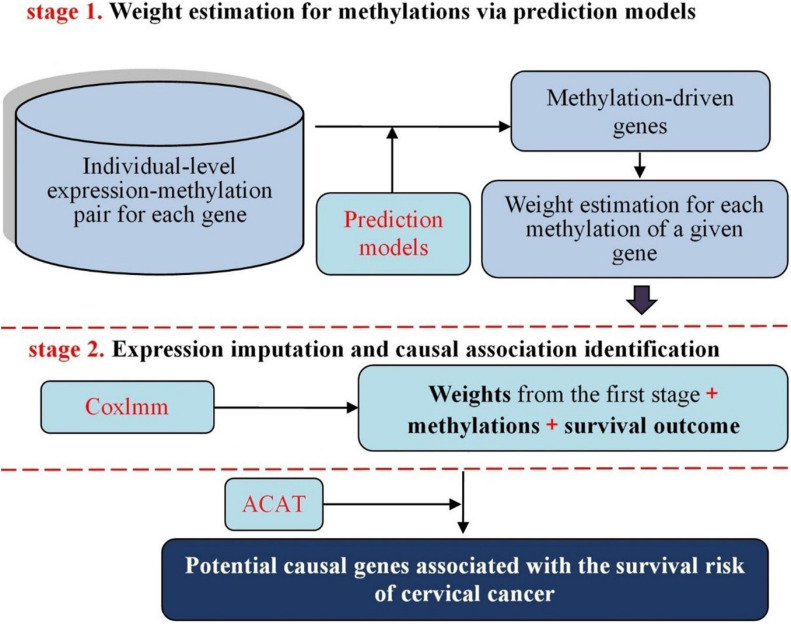
Schematic framework of our proposed two-stage causal inference approach. Top: estimate the weight of each methylation site based on the methylation-expression pair of a given gene with various prediction models; Bottom: evaluate the association between methylation-regulated gene expression (MReX) and the survival of cervical cancer using the Cox linear mixed-effects model and then discover causal genes for cervical cancer in TCGA.

Methodologically, our proposed approach follows the similar principle of prediXcan (Gamazon et al., 2015) that was developed recently to identify causal genes for complex diseases with genetic variants serving as instrumental variables in the framework of MR and transcriptome-wide association studies (TWAS) (Gamazon et al., 2015; Gusev et al., 2016; Zeng and Zhou, 2017; Barbeira et al., 2019; Hu et al., 2019; Wainberg et al., 2019). Specifically, in our context we implement a two-stage inference procedure (Figure 1): in the first stage, the weights (i.e., effect sizes on expression) of DNA methylation alterations within the promoter region and gene body for individual genes are estimated via genetic prediction models; in the second stage, the methylation-regulated gene expression (MReX) is imputed based on the corresponding prediction model and the potential causal association between the gene and the survival risk of cervical cancer is examined using MReX. More importantly, the two-stage based causal inference can be viewed as a special case of MR analysis from a statistical perspective (Zhu and Zhou, 2020). Furthermore, we consider five commonly used prediction models in the first stage of our two-stage inference procedure and exploit the aggregated Cauchy association test (ACAT) method (Liu et al., 2019; Liu and Xie, 2020; Xiao et al., 2020)—a novel combination strategy that is robust against positive correlation—to take advantage of respective strengths of these models while accounting for dependency among the *p*-values of various models.

We finally apply the proposed approach to the cervical cancer dataset in The Cancer Genome Atlas (TCGA) project (Hoadley et al., 2018). A total of 14 potential causal genes were discovered to be associated with the survival risk of cervical cancer when the five prediction models were separately implemented. The total number of potential causal genes was brought to 23 when conducting the combination test with ACAT. Some of the newly discovered genes were reported in previous literature and differentially expressed between tumor and normal tissues. In addition, functional analyses showed that these genes were enriched in tumor-associated pathways.

## Materials and Methods

### TCGA Cervical Cancer Data Sets and Quality Control

**FIGURE 2:**
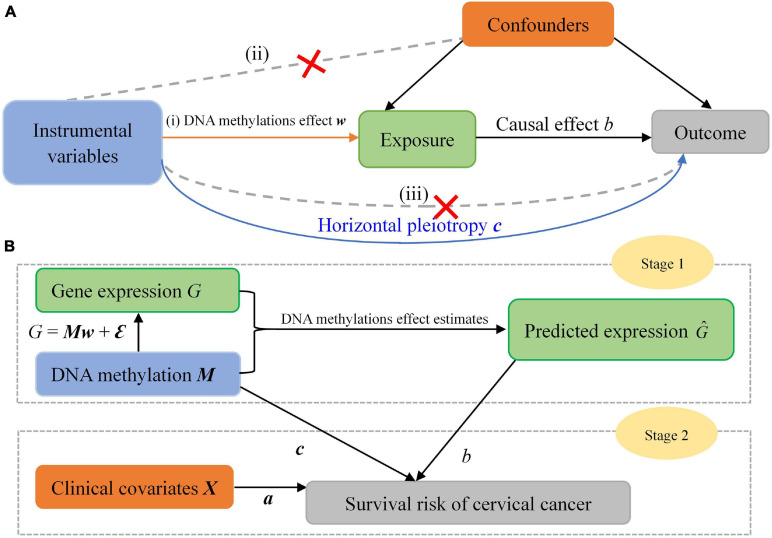
**(A)** Mendelian randomization framework for the two-stage association analysis. The three assumptions for valid instrumental variables (IV) are indicated by arrows or the absence of arrows: (i) the relevance assumption: the IV is robustly associated with the exposure; (ii) the independence assumption: the IV is not associated with confounding factors; (iii) the exclusion restriction assumption: there is no alternative way that the IV affects the outcome other than via the exposure. The blue solid line represents direct effects of DNA methylations. **(B)** Statistical scheme of our two-stage inference approach.

Our analysis mainly relied on publicly available datasets of cervical cancer in TCGA (Hoadley et al., 2018). From https://xenabrowser.net/hub/, we obtained clinical features on 317 samples, 20,530 RSEM normalized expressions on 308 samples, and 485,577 DNA methylation alterations on 312 samples. To avoid racial heterogeneity in survival, expressions, and methylations, when carrying out quality control before the formal analysis, we only reserved 216 white patients. Afterward, we further deleted eight patients for whom gene expressions or methylations cannot be available. We also removed another eight patients who had incomplete clinical covariates. The description of important characteristics of this cervical cancer dataset after filtering is summarized in Table 1. According to the TCGA annotation mapping file, we only considered protein-coding genes and defined in our analysis methylations as those within the gene body and an extended region before the transcription starting site so that the promoter can be included. Then, each gene expression was quantile-transformed so that it followed a standard normal distribution and each methylation was standardized. The missing DNA methylations values and gene expressions (no more than 10%) were simply imputed with median. The flowchart for our study is shown in Figure 2.

**TABLE 1:** Summary information of clinical characteristics for cervical cancer available from TCGA.

Characteristic	Cervical cancer (%)
**Quality control**	
Sample	190
Gene	13,700
Methylation	485,577
Censored	127 (40.06)
Average age (range)	47.8 (20–81)
**Clinical stage (%)**	
T1	109 (57.4)
T2	42 (22.1)
T3	29 (15.3)
T4	10 (5.3)
**Tumor status (%)**	
T0	138 (72.6)
T1	52 (27.4)
Survival time (median/range) (month)	37.7; 0–213.6
**Survival status (%)**	
Living	141 (74.2)
Dead	49 (25.8)

*We conducted a series of quality control for those raw datasets: removed 101 non-white samples, five samples that were not primary solid tumor, 18 samples with missing values in clinical information, and three samples with no gene expressions or DNA methylations. A total of 20,530 genes were originally included, among which 17,611 were protein-coding genes; 3,911 genes were removed because the number of matched methylations for those genes was less than 10.*

### A Two-Stage Inference Approach

#### Linear Models Predicting Gene Expression With DNA Methylation Alterations

We now explain our two-stage inference model (Figure 1). Let *G* be an *n*-vector of gene expression levels for the *i*^*th*^ gene measured on *n* individuals, and ***M*** be an *n* × *p* matrix for a group of DNA methylations that are located within this gene; note that *p* varies gene by gene. In the first stage, we apply the following linear model to link *G* and ***M***

(1) $$G=\mathrm{Mw}+\varepsilon ,\varepsilon ∼N\,\left(0,\sigma_{\varepsilon }^{2}\,{\text{I}}_{n}\right)$$

where ***w*** is a *p*-vector for effect sizes of DNA methylations, ε is an *n*-vector of residual errors following an independent and identical normal distribution with mean zero and variance $\sigma_{\varepsilon }^{2}$, and **I***_*n*_* denotes the *n*-dimensional identity matrix. Because of the possible high-dimensional issue where the number of DNA methylations *p* is larger than the sample size *n*, the commonly used least-squares method is no longer applicable for estimating ***w***. We instead employ several novel prediction models which are specially designed for high-dimensional datasets and particularly consider five regressions including linear mixed-effects model (LMM) (Yang et al., 2010; Makowsky et al., 2011; Zeng et al., 2017), Bayesian sparse linear mixed-effects model (BSLMM) (Zhou et al., 2013), latent Dirichlet process regression (DPR) (Zeng and Zhou, 2017), Lasso (Tibshirani, 1996), and elastic net (ENET) (Zou and Hastie, 2005). Among these methods, LMM, BSLMM, and DPR explicitly incorporate all DNA methylations into the model by assuming diverse prior distributions for the effect sizes, while Lasso and ENET only include some most important DNA methylations with the way of regularization based on variable selection. The details of these models are described in Zhu and Zhou (2020) and Zeng et al. (2021). We implement LMM and BSLMM with the GEMMA software (version 0.94), DPR with the DPR software (Zeng and Zhou, 2017), and Lasso and ENET with the R glmnet package (version 2.0-18) (Friedman et al., 2010). Using these models, we can obtain the estimate of effect sizes of DNA methylations (denoted by $\hat{w}$) as well as the MReX level $\hat{G}$ = $M\,\hat{w}$ for each gene.

#### Cox Mixed-Effect Regression Discovering Methylation-Regulated Genes

In the second stage, we investigate the association between the gene and the survival risk of cervical cancer using the Cox model (Cox, 1972). Besides the direct gene effect based on MReX $\hat{G}$, we also incorporate the impact of DNA methylation alterations into the survival model to explain possible horizontal pleiotropy (Bowden et al., 2015, 2016; Burgess and Thompson, 2017; Slob et al., 2017; Barfield et al., 2018; Verbanck et al., 2018)

(2) $$\frac{h\,\left(t|X,\hat{G},M\right)}{h_{0}\,\left(t\right)}=\exp\left(\mathrm{Xa}+\hat{G}\times b+\mathrm{Mc}\right),c∼N\,\left(0,\sigma_{c}^{2}\right)$$

where *t* is the observed survival time, *h*_0_(*t*) is an arbitrary baseline hazard function, and ***a*** = (*a*_1_, *a*_2_, …, *a*_*m*_) is an *m*-vector of effect sizes for available covariates ***X***, such as age of onset, clinical stage, and tumor status (Table 1); note that, as methylation is highly associated with age, we here explicitly adjust for age as a covariate; *b* is the effect size for the given gene and is of our primary interest, and ***c*** = (*c*_1_, *c*_2_, …, *c*_*p*_) is a *p*-vector of effect sizes for DNA methylations. Because of the same reason of high-dimensional problem mentioned before, we assume *c*’s are random effects following a normal distribution with mean zero and variance $\sigma_{c}^{2}$, leading to the Cox linear mixed-effects regression model (denoted by coxlmm) (Therneau et al., 2003). When ***c*** = 0, or equivalently $\sigma_{c}^{2}$ = 0, coxlmm shown in (2) reduces into the general Cox model where only the influence of the methylation-driven gene exists. We fit coxlmm with the R coxme (version 2.2-10) package (Therneau, 2019) via the Laplace approximation algorithm based on the second-order Taylor series expansion (Therneau et al., 2003). The significance of MReX is examined through the Wald test (*H*_0_: *b* = 0): $Z=\hat{b}/\sqrt{v\,a\,r\,\left(\hat{b}\right)}$, where $\hat{b}$ is the estimate of the effect size *b*, with $v\,a\,r\,\left(\hat{b}\right)$ the variance of the estimate $\hat{b}$. The *p*-values of the *Z* statistic can be easily obtained because it asymptotically follows a standard normal distribution.

#### Three Remarks for the Proposed Two-Stage Causal Inference Method

First, it needs to highlight that instrumental variables are often obtained from external independent datasets in traditional MR studies, leading to the so-called two-sample analysis. However, sometimes, if we have only one sample dataset with individual-level methylations, expressions, and survival outcomes, we can still perform a one-sample MR analysis. In brief, there are two ways to conduct such analysis. First, as done in the present work, one can estimate effect sizes of instrumental variables and examine the association with all individuals. Second, one can split the dataset into two parts, with one part for estimating effect sizes of instrumental variables and the other part for analyzing association between the gene and the outcome. Both the ways have advantages and limitations. Specifically, the major advantage of the first way is that no random split is needed and it has relatively higher power because of larger samples employed. The limitation is that it might suffer from inflation in controlling type I error; however, the resulting inflation is acceptable in terms of recent simulations (Xue et al., 2020). The advantage of the second way is that it can maintain calibrated type I error control, but its power might be limited as smaller samples applied in the estimation stage and the association stage. Another limitation of the second way is that its performance may greatly rely on how to split the dataset. Therefore, in our work we perform our one-sample analysis using all available cervical cancer patients in both stages.

**FIGURE 3:**
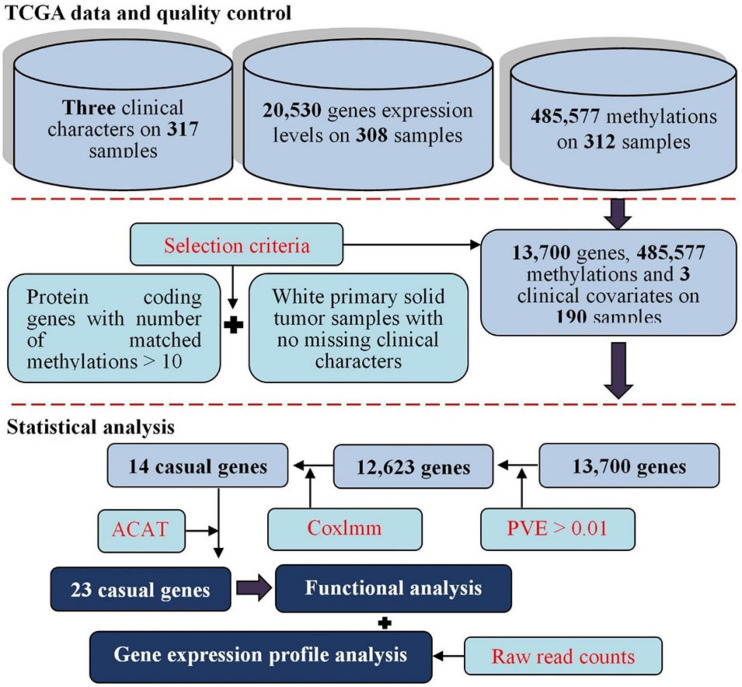
Flowchart for the present study with datasets of cervical cancer available from TCGA. First, various levels of raw datasets were included for cervical cancer; we conducted a series of quality control for those raw datasets. Second, gene expressions predicted with methylations were generated with diverse prediction models, the Cox linear mixed-effects model was applied to identify methylation-driven genes based on predicted expression levels; we aggregated the *p*-values of genes from different prediction models through a *p*-values combination manner to find significant genes that were related to the survival of cervical cancer. Finally, we further implemented functional and differential expression analyses for newly identified associated genes.

Second, in our analysis we apply a group of local methylations serving as instrumental variables, which has the potential of higher power because of more variation of expression explained compared to the strategy of applying only a few significantly gene-associated ones (Zeng and Zhou, 2017; Zeng et al., 2021). In addition, utilizing local methylation CpG sites for a given gene is also widely seen in gene-based statistical genomics analysis when involving methylations (Kingsley et al., 2016; Loucks et al., 2016; Chu and Huang, 2017; Huang, 2019; Liu et al., 2020). However, it has been widely warned in MR studies that incorporating more instrumental variables (e.g., methylations) may have higher risk in violating the third MR assumption (the exclusion restriction assumption; Figure 3) due to unknown biological pathways (Zeng and Zhou, 2019a,b; Zeng et al., 2019; Yuan et al., 2020; Liu et al., 2021). More specifically, methylations themselves might have substantial impact on survival risk through horizontal pleiotropy besides the indirect influence via the pathway of gene. To handle this problem, we attempt to remove possible pleiotropic effects of methylations by adding a random-effect term of methylations in the Cox model. It has been shown that doing this is an effective manner to account for instrumental pleiotropy (Yuan et al., 2020; Liu et al., 2021).

Third, as mentioned before and shown in Figure 3, we construct our two-stage-based causal inference under the framework of MR and TWAS (Yuan et al., 2020; Zhu and Zhou, 2020; Liu et al., 2021; Zeng et al., 2021); therefore, in terms of the principles of the two methods, we possess the potential for identifying putatively causal genes associated with the survival risk of cervical cancer.

### Aggregated Cauchy Association Test

Because multiple prediction models are applied, for each gene we thus yield a set of *p*-values *p*_*k*_ (*k* = 1, 2, …, *K*; with *K* the number of the prediction models) according to (2). Unfortunately, the simple and commonly used Fisher’s method for aggregating mutually independent multiple tests cannot be exploited due to highly positive correlation among individual tests since they are implemented for the same data set with the similar logic (Fisher, 1934; Rice, 2010). To effectively address the aforementioned difficulty of dependency, we apply the recently developed aggregated Cauchy association test (ACAT) (Liu et al., 2019; Liu and Xie, 2020). Specifically, suppose there are a set of *p*-values for each gene and each *p*_*k*_ is uniformly distributed between 0 and 1 under the null; we have

(3) $$\begin{array}{l} p_{ACAT}=\frac{1}{2}-arc\tan\left\{T_{ACAT}/(\overset{K}{\underset{k=\;1}{\sum }}{\text{ω}}_{k})\right\}/\text{π},\\ T_{ACAT}=\overset{K}{\underset{k=\;1}{\sum }}{\text{ω}}_{k}\tan\left\{(\frac{1}{2}-p_{k})\text{π}\right\} \end{array}$$

where *ω_*k*_* represents the nonnegative weight for each *p*_*k*_ with $\sum_{k=1}^{K}\omega_{k}=1$ and *K* = 5; in the absence of prior knowledge, the equal weights are adapted, and assume that *ω_*k*_* is not related to *p*_*k*_. It has been theoretically demonstrated that the dependency among *p*-values imposes little influence on the final pooled *p*-values in ACAT, especially on exceedingly small *p*-values which are of particular interest for practitioners (Li et al., 2019; Liu et al., 2019). Therefore, ACAT renders the potential to allow us to aggregate correlated *p*-values obtained from multiple tests into a single well-calibrated *p*-value that can maintain the type I error control correctly.

### Functional Analysis and Differential Expression Analysis for Newly Identified Associated Genes

Using the proposed two-stage causal inference model, we identified multiple candidate causal genes associated with the survival risk of cervical cancer. We here implemented additional bioinformatics analyses to study their biological functions. First, gene ontology (GO) and Kyoto Encyclopedia of Genes and Genomes (KEGG) pathway analyses were conducted using the R clusterProfiler package (version 3.16.0) (Yu et al., 2012). In addition, to further evaluate the expression profiles of these newly discovered genes, we performed differential expression analysis with 190 cervical tumors and three normal tissues that were also available from TCGA. After normalization with the trimmed mean of M values (TMM) method, differential expressed genes (DEGs) were screened via the exact test based on quantile-adjusted conditional maximum likelihood estimation (Robinson and Smyth, 2008; Li et al., 2013) implemented in the edgeR package (version 3.30.3) (Robinson et al., 2010; McCarthy et al., 2012). Following previous work (Deng et al., 2019), DEGs were defined if false discovery rate (FDR) < 0.05 and | log_2_FC| ≥ 1.0.

## Results

### Cervical Cancer Datasets in TCGA and Methylation-Regulated Genes

After quality control, we reserved 485,577 DNA methylation CpG sites, three clinical covariates (i.e., age of onset, clinical stage, and tumor status) and up to 190 cervical cancer patients of European ancestry. To avoid numerical instability, we focused on protein-coding genes which had at least 10 methylations within the promoter region and the total gene body (Liu et al., 2020). We also first performed the LMM analysis (Visscher et al., 2008; Yang et al., 2011; Zhou et al., 2013) for each protein-coding gene based on its methylations and selected genes with the phenotypic variance explained by methylations larger than 1% (corresponding to a correlation coefficient of 10%). The remaining 12,623 genes are referred to as methylation-regulated genes and included in our subsequent analyses (Figure 2). Most of the genes analyzed (92.0%) have the number of DNA methylation CpG sites less than 50 (Supplementary Figure 1).

### Identification of Potential Causal Genes With Cox Linear Mixed-Effect Regression

**FIGURE 4:**
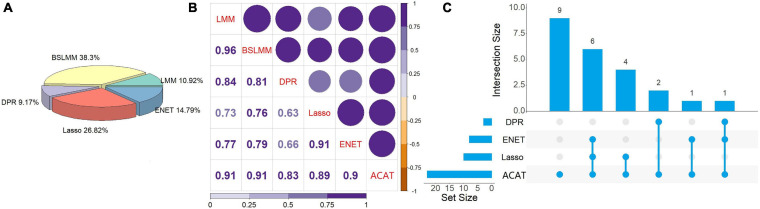
**(A)** The number of prediction models that have maximum *R*^2^ across all the genes analyzed when predicting expression level with using methylations. **(B)** Pearson’s correlation of the *p*-values (in a scale of -log10) obtained via in the Cox linear mixed-effects model with five different prediction models. In the plot the intensity of the color and the size of the circle represent the magnitude of the correlation. **(C)** UpSet plot to illustrate the intersection of associated genes identified by tests with five prediction models. LMM, Linear mixed model; BSLMM, Bayesian sparse linear mixed model; DPR, Latent Dirichlet Process Regression; ENET, elastic net; ACAT, aggregated Cauchy association test.

We employed the coxlmm (Therneau et al., 2003) with various prediction models to examine the relationship between MReX and the survival risk of cervical cancer patients while adjusting for the direct effect of methylations and the confounding effect of clinical covariates. First, we observe that these prediction models display varying performances across genes (Figure 4A). Specifically, some prediction models have higher prediction accuracy for some genes but behave less satisfactorily for others. For example, in terms of *R*^2^, BSLMM behaves well for 38.3% genes (=4,834/12,623), while Lasso, ENET, LMM, and DPR have higher *R*^2^ for 26.82% (=3,386/12,623), 14.79% (=1,867/12,623), 10.92% (=1,378/12,623), and 9.17% (=1,158/12,623) genes, respectively. As expected, the resulting *p*-values of these prediction methods in coxlmm are highly correlated (Figure 4B). For example, the Pearson’s correlation of the *p*-values (in a scale of -log10) ranges from 0.63 between DPR-coxlmm and Lasso-coxlmm to 0.96 between LMM-coxlmm and BSLMM-coxlmm.

**FIGURE 5:**
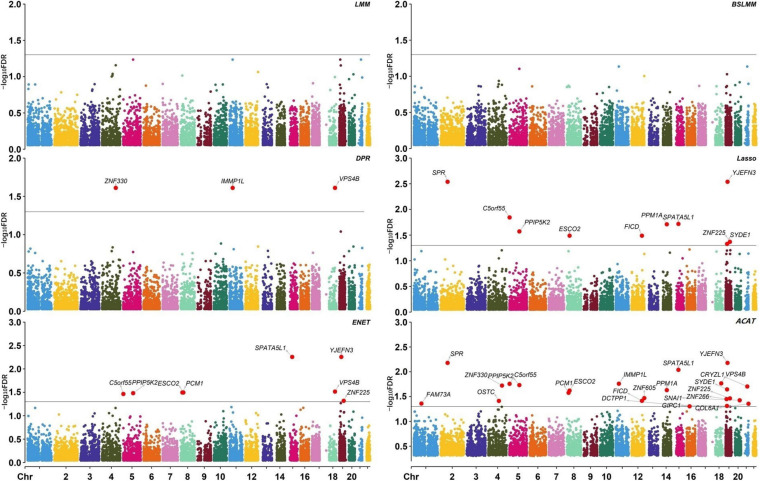
Manhattan plot showing the significance of all genes. Each plot is in a -log10 [false discovery rate (FDR)] scale. Genes with -log10 FDR > 1.3 (i.e. FDR < 0.05) are highlighted. DPR, Latent Dirichlet Process Regression; ENET, elastic net; ACAT, aggregated Cauchy association test.

Based on the results of coxlmm, a total of 14 unique potential associated genes (FDR < 0.05) are identified (Table 2). Specifically, we detect three genes with DPR-coxlmm, 10 genes via Lasso-coxlmm, and eight genes through ENET-coxlmm but do not discover any potential associated genes using LMM-coxlmm or BSLMM-coxlmm (Figure 5). Among these, six genes (i.e., *YJEFN3*, *SPATA5L1*, *C5orf55*, *PPIP5K2*, *ESCO2*, and *ZNF225*) are simultaneously found by Lasso-coxlmm and ENET-coxlmm, while only one gene (i.e., *VPS4B*) is simultaneously discovered by DPR-coxlmm and ENET-coxlmm (Figure 4C).

**TABLE 2:** A total of 23 associated genes identified via the proposed two-stage causal inference approach.

Gene	CHR	Position	*p*-values						Effect size				
			LMM	BSLMM	DPR	Lasso	ENET	ACAT	LMM	BSLMM	DPR	Lasso	ENET
**Discovered by the two-stage inference with a single prediction model**													
*SPR*	2	73,089,746–73,122,092	0.795	0.820	0.890	0.003	0.791	0.007	−0.157	−0.161	−0.167	0.854	−0.216
YJEFN3*	19	19,637,360–19,648,336	0.848	0.877	0.936	0.003	0.006	0.007	0.065	0.045	0.010	−0.800	−0.800
SPATA5L1*	15	45,693,741–45,699,035	0.789	0.828	0.888	0.019	0.006	0.009	0.159	0.139	0.155	0.777	0.742
*VPS4B*	18	61,057,089–61,090,282	0.102	0.242	0.024	0.177	0.031	0.017	−0.637	−0.610	−0.779	−0.631	−0.772
IMMP1L*	11	31,464,983–31,531,760	0.058	0.073	0.024	0.623	0.343	0.017	−0.508	−0.510	−0.599	−0.314	−0.440
C5orf55*	5	440,158–444,229	0.704	0.684	0.825	0.014	0.034	0.018	−0.317	−0.326	−0.310	−0.657	−0.704
PPIP5K2*	5	102,455,924–102,548,824	0.058	0.079	0.169	0.027	0.033	0.019	−0.550	−0.574	−0.519	−0.521	−0.496
ZNF330*	4	142,141,696–142,155,292	0.070	0.132	0.024	0.162	0.146	0.019	−0.577	−0.549	−0.695	−0.516	−0.621
*SYDE1*	19	15,217,634–15,227,220	0.070	0.094	0.091	0.047	0.054	0.023	0.799	0.787	0.779	0.799	0.809
PPM1A*	14	60,711,064–60,761,525	0.347	0.331	0.438	0.020	0.328	0.023	−0.775	−0.801	−0.724	−0.848	−0.784
ESCO2*	8	27,630,985–27,661,590	0.254	0.141	0.323	0.033	0.032	0.024	−0.618	−0.678	−0.597	−0.778	−0.787
*PCM1*	8	17,779,996–17,886,268	0.097	0.136	0.180	0.066	0.032	0.027	−0.610	−0.530	−0.552	−0.624	−0.673
ZNF225*	19	44,617,124–44,620,505	0.241	0.273	0.282	0.043	0.048	0.034	−0.283	−0.278	−0.283	−0.324	−0.324
FICD*	12	108,851,555–108,920,535	0.759	0.828	0.879	0.033	0.264	0.038	−0.154	−0.116	−0.135	−0.638	−0.534
**Newly discovered by ACAT which combines results obtained from various prediction models**													
CRYZL1*	21	34,962,101–35,015,323	0.058	0.073	0.379	0.173	0.315	0.020	−0.673	−0.674	−0.540	−0.586	−0.488
ZNF605*	12	133,518,352–133,536,293	0.086	0.099	0.143	0.074	0.097	0.034	−0.480	−0.486	−0.452	−0.471	−0.444
ZNF266*	19	9,517,603–9,577,375	0.058	0.59	0.680	0.733	0.568	0.035	−0.479	−0.432	−0.511	−0.323	−0.433
*SNAI1*	20	48,595,808–48,629,343	0.093	0.121	0.143	0.074	0.082	0.037	0.614	0.596	0.597	0.579	0.578
OSTC*	4	109,571,401–109,588,819	0.092	0.116	0.147	0.090	0.100	0.039	−0.539	−0.535	−0.551	−0.510	−0.509
*FAM73A*	1	78,244,204–78,343,254	0.129	0.145	0.174	0.065	0.068	0.044	0.585	0.561	0.590	0.872	0.741
*COL6A1*	21	47,361,643–47,469,105	0.103	0.127	0.150	0.073	0.110	0.044	0.635	0.583	0.604	0.634	0.619
*GIPC1*	19	14,589,147–14,608,093	0.144	0.137	0.372	0.063	0.080	0.049	−0.655	−0.677	−0.553	−0.703	−0.697
*DCTPP1*	16	30,430,088–30,442,010	0.546	0.884	0.785	0.060	0.068	0.050	−0.284	0.028	−0.222	0.633	0.633

*In the table, we show FDR and the effect size for each gene. CHR, chromosome; LMM, linear mixed model; BSLMM, Bayesian sparse linear mixed model; DPR, Latent Dirichlet Process Regression; ENET, elastic net; ACAT, aggregated Cauchy association test. Position is built under hg19. The genes with asterisk are viewed as new novel genes (i.e., *YJEFN3*, *SPATA5L1*, *IMMP1L*, *C5orf55*, *PPIP5K2*, *ZNF330*, *PPM1A*, *ESCO2*, *ZNF225*, *FICD*, *CRYZL1*, *ZNF605*, *ZNF266*, and *OSTC*).*

Among these potential associated genes, we find that *PCM1* (FDR_ENET_ = 0.032), classified to the cell cycle control network, was previously discovered to be associated with the early stage of cervical cancer (Güzel et al., 2018). *SPR* (FDR_Lasso_ = 0.003) is located within the 1-Mb genetic region of previous GWAS-identified gene *ALMS1* (Masuda et al., 2020). In addition, *VPS4B* (FDR_DPR_ = 0.024 and FDR_ENET_ = 0.031) is a subtype of *VPS4* which is the component of the *ESCRT* machinery and plays an essential role in HPV infectious entry and capsid disassembly (Broniarczyk et al., 2017). The remaining 10 genes (i.e., *YJEFN3*, *SPATA5L1*, *C5orf55*, *PPM1A*, *IMMP1L*, *ZNF330*, *PPIP5K2*, *ESCO2*, *FICD*, and *ZNF225*) are not directly reported to be related to the survival risk of cervical cancer in previous literature. However, for these genes we find suggestive indirect evidence that may support their association with the survival risk of cervical cancer. Specifically, for example, *YJEFN3* is a member of the human *YJEFN* domain-containing protein family strongly expressing in Leydig cell tumors and in the fibromas and participates in cholesterol processing and steroid hormone metabolism (Rudolph et al., 2007). *SPATA5L1* might play a key role in inhibiting ATP hydrolysis and four-way junction helicase activity and further influence DNA replication and pathogenesis (White et al., 2005; Rudolph et al., 2007). Smac/DIABLO was expressed *de novo* in a certain subset of cervical tumors (Martinez-Ruiz et al., 2008), while mature Smac/DIABLO was produced on the mitochondrial inner membrane via *IMMP1L* (Burri et al., 2005). *PPIP5* kinases (e.g., *PPIP5K2*) mediate PP-IP binding, activate casein kinase 2 (CK2), and promote the phosphorylation of the TTT complex, which stimulates DNA-PK/ATM to activate p53 on the cancer cells (Fridy et al., 2007; Lee et al., 2020). There exists evidence that miR-135b leads to cervical cancer cell transformation (Leung et al., 2014) and downregulated miR-135b expression could inhibit the proliferation and invasion of tumor cells by upregulating *PPM1A* (Gao et al., 2019).

### ACAT Combining *p*-Values From Different Prediction Models

As mentioned before, because the *p*-values obtained from coxlmm with diverse prediction models are highly dependent (Figure 4B), we effectively apply ACAT to combine the five *p*-values and generate an overall significance for each gene (Figure 3 and Table 2). Nine associated genes are additionally discovered (Figure 4C), including *CRYZL1*, *ZNF605*, *ZNF266*, *SNAI1*, *OSTC*, *FAM73A*, *COL6A1*, *GIPC1*, and *DCTPP1*. We found 87.0% (=20/23; except *SPR*, *YJEFN3*, and *DCTPP1*) directions of gene effect consistent across the five genetic prediction models (Table 2). In addition, for these genes it seems that the association signals are mostly driven by LASSO and ENET (Table 2). This observation might imply that there may be only a few of methylations implicated in regulating the expression levels of these genes. As a result, sparse prediction models (i.e., LASSO and ENET) lead to higher power in subsequent association analysis due to better accuracy (Zeng et al., 2021). Among these genes, five (i.e., *SNAI1*, *COL6A1*, *GIPC1*, *DCTPP1*, and *FAM73A*) were identified in prior work and *SYDE1* locates within the 1-Mb generic region of *GIPC1* (Chen et al., 2013a, 2016; Shi et al., 2013; Miura et al., 2014; Leo et al., 2017; Takeuchi et al., 2019; Masuda et al., 2020). Specifically, it is shown that *SNAI1*, along with *ZEB1*, regulated the epithelial–mesenchymal transition and was then involved in the metastasis of cervical cancer (Chen et al., 2013b). The upregulated *COL6A1* expression in the tissues of cervical cancer was related to poor clinical prognosis and treated as an important biomarker of cervical cancer progression (Hou et al., 2016). The downregulation of *GIPC1* in cervical cancer with HPV-18 infection can lead to the resistance to cytostatic transforming growth factor β signaling through *TGFβR3* destabilization (Katoh, 2013).

In addition, *DCTPP1* was found to be differentially expressed in normal and cancerous tissues and it was significantly accumulated in the nucleus of cervical carcinoma, implying the important role of *DCTPP1* under malignant pathology (Zhang et al., 2013). Family with sequence similarity 73, member A (*FAM73A*) is the downregulated gene of DNA from exfoliated cervical cells in terms of the HPV-16 variant analysis (Green, 2019; Meng et al., 2020). *CRYZL1* contains a reduced nicotinamide adenine dinucleotide (phosphate) (*NAD(P)H*) binding site which is involved in cellular metabolism, while cervical lesions are associated with cellular metabolic abnormalities (Wang et al., 2020). It is previously found that the members of the ZNF family interact with nucleic acids, proteins, and small molecules and are involved in a variety of crucial molecular processes in cervical tumor cells at replication, transcriptional, and translational levels. Thus, *ZNF605* and *ZNF266* may be potentially targetable (Das et al., 2016; The Cancer Genome Atlas Research Network, 2017; Li et al., 2018). *OSTC* can regulate gamma-secretase (Wilson et al., 2011) while this secretase affects the ability of HPV pseudo-viruses infection in both human HaCat cells and mouse cells (Huang et al., 2010).

In summary, compared with the tests via individual prediction methods, it is demonstrated that ACAT greatly improves statistical power by combining dependent tests and thus identifies more potential prognosis-associated genes for the survival risk of cervical cancer. Totally, 23 genes are discovered to be related to the survival risk of cervical cancer, among which 14 genes are likely newly novel genes (i.e., *YJEFN3*, *SPATA5L1*, *IMMP1L*, *C5orf55*, *PPIP5K2*, *ZNF330*, *CRYZL1*, *PPM1A*, *ESCO2*, *ZNF605*, *ZNF225*, *ZNF266*, *FICD*, and *OSTC*).

### Identification of DEGs, GO, and KEGG Pathway Annotation

**FIGURE 6:**
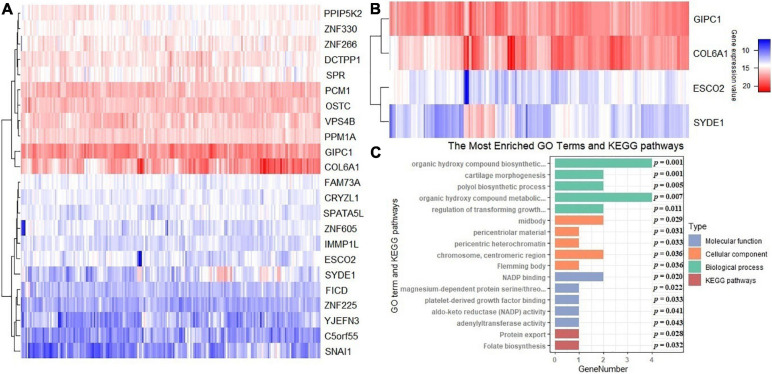
**(A)** Heatmap of expression levels for these 23 newly identified causal genes of cervical cancer. **(B)** Heatmap for differentially expressed genes. **(C)** Gene ontology (GO) and Kyoto Encyclopedia of Genes and Genomes (KEGG) pathway enrichment analyses for the 23 genes; the *p*-values are also shown. Count Number denotes the number of genes related to the enriched GO or KEGG pathway.

In terms of the differential expression analysis, four DEGs are detected among the 23 new potential causal genes identified above (Figure 6A). *COL6A1* and *SYDE1* are upregulated genes, while *ESCO2* and *GIPC1* are downregulated genes (Figure 6B). To explore the potential functions of these genes that may be associated with the tumorigenesis and development of cervical cancer, we performed functional enrichment analysis with GO and KEGG using the R package clusterProfiler (version 3.16.0) (Yu et al., 2012). The top five GO terms of three parts and two KEGG pathways are shown in Figure 6C.

The GO biological process (BP) terms are remarkably enriched in the polyol metabolic process, regulation of biosynthetic process, and signaling pathway and chondrocyte differentiation. For the GO cellular component (CC) terms, the target genes are concentrated in the midbody, pericentriolar material, and so on. The molecular function (MF) category was focused on NADP binding, platelet-derived growth factor binding (Supplementary Table 1). The KEGG enrichment analysis indicates that these genes are remarkably enriched in tumor-associated pathways, including protein export (*P* = 0.028) and folate biosynthesis (*P* = 0.032) (Figure 6C). The combined action of folate biosynthesis and graft-vs.-host disease was demonstrated to be significantly associated with cervical cancer in suit: *HLA-DPB1* (Ivansson et al., 2011). The upregulated differentially expressed genes are mostly associated with cartilage morphogenesis (ontology: BP), collagen trimer (ontology: CC), and extracellular matrix structural constituent conferring tensile strength (ontology: MF). The downregulated differentially expressed genes are mostly associated with organic hydroxy compound biosynthetic process (ontology: BP), organic hydroxy compound metabolic process (ontology: BP), and dendritic shaft (ontology: CC) (Supplementary Table 1). The functional enrichment results suggest that these newly discovered potential causal genes may participate in oncogenicity and tumor progression in cervical cancer through regulating relevant biological processes and critical pathways.

## Discussion

Given the severe health threat among women and little knowledge of genetic basis for cervical cancer, persistent work should be done to discover genes that are causally related to cervical cancer (Chen and Gyllensten, 2015). The present study is one of such efforts with the aim to detect newly possible causal genes for the survival risk of cervical cancer through integrative genomic methods. The two-stage inference analysis pipeline applied in this work can be considered as a gene-centered integration approach by aggregating omics datasets and clinical information. With the growing high-throughput omics datasets in cancer research over recent years (Hoadley et al., 2018), it is well-recognized that the utilization of only one single level of genomic measurements is insufficient to completely untangle the etiology of cancer prognosis (Zhao et al., 2014; Hoadley et al., 2018). Based on the omics datasets of TCGA measured from multiple platforms, we treated the gene expression as the exposure and the survival time as the outcome to explore the possible causal genes of the survival risk of cervical cancer within the framework of the two-stage MR study to avoid the reverse causation.

One critical step in our two-stage inference is to evaluate the effect relationship between a group of DNA methylation CpG sites and the expression level for each gene. The power of the subsequent association performed in coxlmm would greatly depend on how well the prediction model utilized can capture the underlying genetic architecture of the transcriptome (Gamazon et al., 2015; Gusev et al., 2016; Zeng and Zhou, 2017; Barbeira et al., 2019; Hu et al., 2019; Wainberg et al., 2019), which can differ in the numbers, effect sizes, and effect directions of causal methylation alterations in diverse genes. Therefore, a powerful two-stage inference approach should in the first stage choose a prediction model whose prior effect distribution closely matches the true effect distribution so that it can approximate well the genetic architecture of the gene (Zhou et al., 2013; Zeng and Zhou, 2017; Zhu and Zhou, 2020). For example, if DNA methylation alterations have effect sizes following a normal distribution, then LMM-coxlmm would be more powerful; on the other hand, if only a very small fraction of DNA methylation alterations may be predictive for the gene expression, then the test with sparse prediction models (e.g., Lasso-coxlmm and ENET-coxlmm) would be superior. Due to unknown true association patterns, there is no uniformly most powerful test. As a result, the two-stage association test may perform well for one gene, but not necessarily for another.

To leverage the advantage of distinct prediction models to improve power, instead selecting an optimal prediction model, in the present study we considered a wide range of prediction models in our two-stage inference procedure. It can be imaged that the resulting *p*-values would be highly correlated because they are generated with the same data set following the similar logic (Figure 3). The correlation structure of these *p*-values also depends on the true architecture of gene expression, which however, is rarely known in advance and is likely to vary from one gene to another across the genome. Therefore, it is desirable to construct an omnibus test that integrates the advantage of multiple prediction approaches and is robust against distinct transcriptomic architectures. To achieve this aim, we exploited ACAT (Liu et al., 2019; Liu and Xie, 2020) to combine these correlated *p*-values and integrating individual strengths of various tests. As illustrated in our empirical application, ACAT achieves relatively higher power since it aggregates genetic association information across different tests.

Compared to previous similar methods, the proposed two-stage inference approach differs in three aspects. First, unlike prediXcan (Gamazon et al., 2015) we constructed the two-stage inference procedure in one sample, leading to the so-called one-sample two-stage regression (Xue et al., 2020). Second, multiple competing prediction models rather than a single model were utilized and combined with ACAT which was *p*-value calibrated (Liu et al., 2019; Sun et al., 2020; Xiao et al., 2020). Thus, our strategy often has higher power compared to the test with the single prediction model. Third, due to widespread pleiotropic effects in omics (Bowden et al., 2015, 2016; Burgess and Thompson, 2017; Slob et al., 2017; Barfield et al., 2018; Verbanck et al., 2018), we also considered the direct influence of methylations. Therefore, our results would be robust against the bias of pleiotropy of instrumental variables that are commonly encountered in MR.

However, the present study is not without limitation. First, these newly identified methylation-regulated genes were detected only in TCGA; no external relevant expression profiles were applied for validation. Second, we only employed methylations as instrumental variables; other omic measurements that regulate gene expression (e.g., genetic variants; Manor and Segal, 2013, 2015; Zeng et al., 2017) can be also simultaneously incorporated to further improve power. Third, we only utilized local methylation CpG sites of a gene as candidate instruments. It is not known whether the power can be further enhanced if the global methylation CpG sites are exploited. Fourth, the present study assumed a linear relationship for each methylation–expression pair. While a linear relationship can be methodologically interpreted as a first-order approximation to nonlinear relationship (Zhou et al., 2013), modeling a linear relationship may be suboptimal and suffer from power loss if the true relationship is nonlinear. Fifth, due to the complicated standard error structures for those prediction models, in terms of the assumption of no measurement error (NOME) (Bowden et al., 2016), we did not incorporate the uncertainty in the estimated effect sizes of methylations into our two-stage approach, although such uncertainty may be important in integrative genomic causal inference (Yeung et al., 2019; Yuan et al., 2020). Actually, we note that many previous two-stage MR studies or TWAS approaches followed this NOME principle (Gamazon et al., 2015; Bowden et al., 2016; Gusev et al., 2016; Zeng and Zhou, 2017).

## Conclusion

In summary, using the proposed two-stage causal inference approach within the framework of MR analysis, we discovered a total of 14 potential causal genes which were associated with the survival risk of cervical cancer patients when separately applying five commonly used prediction models. The number of possible causal genes was brought to 23 when employing the combination method of ACAT. Some of these genes were differentially expressed between tumor and normal tissue and were enriched in tumor-associated pathways. Our findings provide new insights into the genetic etiology of the survival risk of cervical cancer and suggest possibly potential therapeutic targets for cervical cancer in the future.

## Data Availability Statement

The original contributions presented in the study are included in the article/Supplementary Material, further inquiries can be directed to the corresponding author/s.

## Author Contributions

PZ conceived the idea for the study. PZ, JZ, HL, FG, TW, and SZ obtained the data and performed the data analyses. PZ, JZ, HL, HZ, and SZ interpreted the results of the data analyses. PZ, FG, JZ, and HL wrote the manuscript with the participation of all authors.

## Conflict of Interest

The authors declare that the research was conducted in the absence of any commercial or financial relationships that could be construed as a potential conflict of interest.

## Abbreviations

BP: biological process
BSLMM: Bayesian sparse linear mixed model
Coxlmm: Cox linear mixed-effect model
CC: cellular component
DEG: differential expressed genes
DPR: latent Dirichlet Process Regression
EBI: the European Bioinformatics Institute
ENET: elastic net
FDR: false discovery rate
GO: Gene Ontology
GWAS: genome-wide association study
ACAT: aggregated Cauchy association test
HPV: human papillomavirus
KGEE: Kyoto Encyclopedia of Genes and Genomes
LMM: Linear mixed model
MF: molecular function
MR: Mendelian randomization
TCGA: The Cancer Genome Atlas
TWAS: transcriptome-wide association study.
