# Supplementary material for: Leveraging Methylation Alterations to Discover Potential Causal Genes Associated With the Survival Risk of Cervical Cancer in TCGA Through a Two-Stage Inference Approach
Source: Front Genet. 2021 Jun 2;12:667877. doi: 10.3389/fgene.2021.667877 (PMC8206792; doi:10.3389/fgene.2021.667877)
Supplement: Supplementary file 1 [file Data_Sheet_1.docx]

Supplementary Material

# Supplementary Tables

Table S1. Functional term enrichment analysis by casual genes

| GOID | Ontology | Description | P | Gene | GOID | Ontology | Description | *P* | Gene |
| --- | --- | --- | --- | --- | --- | --- | --- | --- | --- |
| GO:1901617 | BP | organic hydroxy compound biosynthetic process | 0.001 | *GIPC1/PPIP5K2*  *SNAI1/SPR* | GO:0097734 | BP | extracellular exosome biogenesis | 0.034 | *VPS4B* |
| GO:0060536 | BP | cartilage morphogenesis | 0.001 | *COL6A1/SNAI1* | GO:0140112 | BP | extracellular vesicle biogenesis | 0.034 | *VPS4B* |
| GO:0046173 | BP | polyol biosynthetic process | 0.005 | *PPIP5K2/SPR* | GO:0010389 | BP | regulation of G2/M transition of mitotic cell cycle | 0.034 | *PCM1/VPS4B* |
| GO:1901615 | BP | organic hydroxy compound metabolic process | 0.007 | *GIPC1/PPIP5K2*  */SNAI1/SPR* | GO:0003272 | BP | endocardial cushion formation | 0.036 | *SNAI1* |
| GO:0017015 | BP | regulation of transforming growth factor beta receptor signaling pathway | 0.011 | *GIPC1/PPM1A* | GO:0006582 | BP | melanin metabolic process | 0.036 | *GIPC1* |
| GO:1903844 | BP | regulation of cellular response to transforming growth factor beta stimulus | 0.011 | *GIPC1/PPM1A* | GO:0010894 | BP | negative regulation of steroid biosynthetic process | 0.036 | *SNAI1* |
| GO:0001704 | BP | formation of primary germ layer | 0.012 | *COL6A1/SNAI1* | GO:0031365 | BP | N-terminal protein amino acid modification | 0.036 | *PPM1A* |
| GO:0007098 | BP | centrosome cycle | 0.013 | *PCM1/VPS4B* | GO:0036258 | BP | multivesicular body assembly | 0.036 | *VPS4B* |
| GO:0019751 | BP | polyol metabolic process | 0.013 | *PPIP5K2/SPR* | GO:0051647 | BP | nucleus localization | 0.036 | *PCM1* |
| GO:0031023 | BP | microtubule organizing center organization | 0.014 | *PCM1/VPS4B* | GO:0090169 | BP | regulation of spindle assembly | 0.036 | *VPS4B* |
| GO:0006244 | BP | pyrimidine nucleotide catabolic process | 0.017 | *DCTPP1* | GO:1900101 | BP | regulation of endoplasmic reticulum unfolded protein response | 0.036 | *FICD* |
| GO:0042559 | BP | pteridine-containing compound biosynthetic process | 0.017 | *SPR* | GO:0051216 | BP | cartilage development | 0.037 | *COL6A1/SNAI1* |
| GO:0061314 | BP | Notch signaling involved in heart development | 0.017 | *SNAI1* | GO:0003413 | BP | chondrocyte differentiation involved in endochondral bone morphogenesis | 0.038 | *COL6A1* |
| GO:1900376 | BP | regulation of secondary metabolite biosynthetic process | 0.017 | *GIPC1* | GO:0009110 | BP | vitamin biosynthetic process | 0.038 | *SNAI1* |
| GO:0006020 | BP | inositol metabolic process | 0.019 | *PPIP5K2* | GO:0036257 | BP | multivesicular body organization | 0.038 | *VPS4B* |
| GO:0007183 | BP | SMAD protein complex assembly | 0.019 | *PPM1A* | GO:0042359 | BP | vitamin D metabolic process | 0.038 | *SNAI1* |
| GO:0009143 | BP | nucleoside triphosphate catabolic process | 0.019 | *DCTPP1* | GO:0045939 | BP | negative regulation of steroid metabolic process | 0.038 | *SNAI1* |
| GO:0030656 | BP | regulation of vitamin metabolic process | 0.019 | *SNAI1* | GO:0045992 | BP | negative regulation of embryonic development | 0.038 | *SNAI1* |
| GO:0034312 | BP | diol biosynthetic process | 0.019 | *SPR* | GO:0001649 | BP | osteoblast differentiation | 0.038 | *COL6A1/SNAI1* |
| GO:0060707 | BP | trophoblast giant cell differentiation | 0.019 | *SNAI1* | GO:0017157 | BP | regulation of exocytosis | 0.039 | *GIPC1/VPS4B* |
| GO:0070208 | BP | protein heterotrimerization | 0.019 | *COL6A1* | GO:0010971 | BP | positive regulation of G2/M transition of mitotic cell cycle | 0.039 | *VPS4B* |
| GO:1902188 | BP | positive regulation of viral release from host cell | 0.019 | *VPS4B* | GO:0061311 | BP | cell surface receptor signaling pathway involved in heart development | 0.039 | *SNAI1* |
| GO:1903894 | BP | regulation of IRE1-mediated unfolded protein response | 0.019 | *FICD* | GO:1902749 | BP | regulation of cell cycle G2/M phase transition | 0.040 | *PCM1/VPS4B* |
| GO:0000910 | BP | cytokinesis | 0.020 | *GIPC1/VPS4B* | GO:0003180 | BP | aortic valve morphogenesis | 0.041 | *SNAI1* |
| GO:2000241 | BP | regulation of reproductive process | 0.020 | *SNAI1/SYDE1* | GO:0009394 | BP | 2'-deoxyribonucleotide metabolic process | 0.041 | *DCTPP1* |
| GO:0034311 | BP | diol metabolic process | 0.021 | *SPR* | GO:0060706 | BP | cell differentiation involved in embryonic placenta development | 0.041 | *SNAI1* |
| GO:0042368 | BP | vitamin D biosynthetic process | 0.021 | *SNAI1* | GO:0071985 | BP | multivesicular body sorting pathway | 0.041 | *VPS4B* |
| GO:0043455 | BP | regulation of secondary metabolic process | 0.021 | *GIPC1* | GO:0090092 | BP | regulation of transmembrane receptor protein serine/threonine kinase signaling pathway | 0.042 | *GIPC1/PPM1A* |
| GO:0003414 | BP | chondrocyte morphogenesis involved in endochondral bone morphogenesis | 0.022 | *COL6A1* | GO:0019692 | BP | deoxyribose phosphate metabolic process | 0.043 | *DCTPP1* |
| GO:0003429 | BP | growth plate cartilage chondrocyte morphogenesis | 0.022 | *COL6A1* | GO:0030511 | BP | positive regulation of transforming growth factor beta receptor signaling pathway | 0.043 | *GIPC1* |
| GO:0009200 | BP | deoxyribonucleoside triphosphate metabolic process | 0.022 | *DCTPP1* | GO:0032506 | BP | cytokinetic process | 0.043 | *VPS4B* |
| GO:0061952 | BP | midbody abscission | 0.022 | *VPS4B* | GO:1902186 | BP | regulation of viral release from host cell | 0.043 | *VPS4B* |
| GO:0090171 | BP | chondrocyte morphogenesis | 0.022 | *COL6A1* | GO:1903846 | BP | positive regulation of cellular response to transforming growth factor beta stimulus | 0.043 | *GIPC1* |
| GO:0003422 | BP | growth plate cartilage morphogenesis | 0.024 | *COL6A1* | GO:0044550 | BP | secondary metabolite biosynthetic process | 0.044 | *GIPC1* |
| GO:0009147 | BP | pyrimidine nucleoside triphosphate metabolic process | 0.026 | *DCTPP1* | GO:1902751 | BP | positive regulation of cell cycle G2/M phase transition | 0.044 | *VPS4B* |
| GO:0009219 | BP | pyrimidine deoxyribonucleotide metabolic process | 0.026 | *DCTPP1* | GO:0009262 | BP | deoxyribonucleotide metabolic process | 0.046 | *DCTPP1* |
| GO:0009264 | BP | deoxyribonucleotide catabolic process | 0.026 | *DCTPP1* | GO:0042558 | BP | pteridine-containing compound metabolic process | 0.046 | *SPR* |
| GO:0031069 | BP | hair follicle morphogenesis | 0.026 | *SNAI1* | GO:0098760 | BP | response to interleukin-7 | 0.046 | *GIPC1* |
| GO:0035970 | BP | peptidyl-threonine dephosphorylation | 0.026 | *PPM1A* | GO:0098761 | BP | cellular response to interleukin-7 | 0.046 | *GIPC1* |
| GO:0039702 | BP | viral budding via host ESCRT complex | 0.026 | *VPS4B* | GO:1901661 | BP | quinone metabolic process | 0.046 | *CRYZL1* |
| GO:0042362 | BP | fat-soluble vitamin biosynthetic process | 0.026 | *SNAI1* | GO:1902230 | BP | negative regulation of intrinsic apoptotic signaling pathway in response to DNA damage | 0.046 | *SNAI1* |
| GO:0043162 | BP | ubiquitin-dependent protein catabolic process via the multivesicular body sorting pathway | 0.026 | *VPS4B* | GO:0003176 | BP | aortic valve development | 0.048 | *SNAI1* |
| GO:0043518 | BP | negative regulation of DNA damage response, signal transduction by p53 class mediator | 0.026 | *SNAI1* | GO:0019068 | BP | virion assembly | 0.048 | *VPS4B* |
| GO:0046386 | BP | deoxyribose phosphate catabolic process | 0.026 | *DCTPP1* | GO:0043902 | BP | positive regulation of multi-organism process | 0.048 | *SYDE1* |
| GO:0060972 | BP | left/right pattern formation | 0.026 | *SNAI1* | GO:0071539 | BP | protein localization to centrosome | 0.048 | *PCM1* |
| GO:1901673 | BP | regulation of mitotic spindle assembly | 0.026 | *VPS4B* | GO:0072529 | BP | pyrimidine-containing compound catabolic process | 0.048 | *DCTPP1* |
| GO:1903543 | BP | positive regulation of exosomal secretion | 0.026 | *VPS4B* | GO:1901797 | BP | negative regulation of signal transduction by p53 class mediator | 0.048 | *SNAI1* |
| GO:0046165 | BP | alcohol biosynthetic process | 0.026 | *PPIP5K2/SPR* | GO:0032388 | BP | positive regulation of intracellular transport | 0.048 | *PCM1/PPM1A* |
| GO:0003198 | BP | epithelial to mesenchymal transition involved in endocardial cushion formation | 0.027 | *SNAI1* | GO:0071560 | BP | cellular response to transforming growth factor beta stimulus | 0.049 | *GIPC1/PPM1A* |
| GO:1902410 | BP | mitotic cytokinetic process | 0.027 | *VPS4B* | GO:0003417 | BP | growth plate cartilage development | 0.049 | *COL6A1* |
| GO:1903541 | BP | regulation of exosomal secretion | 0.027 | *VPS4B* | GO:0019076 | BP | viral release from host cell | 0.049 | *VPS4B* |
| GO:0007369 | BP | gastrulation | 0.027 | *COL6A1/SNAI1* | GO:0035890 | BP | exit from host | 0.049 | *VPS4B* |
| GO:0003418 | BP | growth plate cartilage chondrocyte differentiation | 0.029 | *COL6A1* | GO:0035891 | BP | exit from host cell | 0.049 | *VPS4B* |
| GO:0003433 | BP | chondrocyte development involved in endochondral bone morphogenesis | 0.029 | *COL6A1* | GO:0060317 | BP | cardiac epithelial to mesenchymal transition | 0.049 | *SNAI1* |
| GO:0046755 | BP | viral budding | 0.029 | *VPS4B* | GO:1905508 | BP | protein localization to microtubule organizing center | 0.049 | *PCM1* |
| GO:0097150 | BP | neuronal stem cell population maintenance | 0.029 | *PCM1* | GO:0030496 | CC | midbody | 0.029 | *VPS4B/ZNF330* |
| GO:0090316 | BP | positive regulation of intracellular protein transport | 0.030 | *PCM1/PPM1A* | GO:0000242 | CC | pericentriolar material | 0.031 | *PCM1* |
| GO:0007097 | BP | nuclear migration | 0.031 | *PCM1* | GO:0005721 | CC | pericentric heterochromatin | 0.033 | *SNAI1* |
| GO:0032509 | BP | endosome transport via multivesicular body sorting pathway | 0.031 | *VPS4B* | GO:0000775 | CC | chromosome, centromeric region | 0.036 | *SNAI1/ZNF330* |
| GO:0034453 | BP | microtubule anchoring | 0.031 | *PCM1* | GO:0090543 | CC | Flemming body | 0.036 | *VPS4B* |
| GO:0048730 | BP | epidermis morphogenesis | 0.031 | *SNAI1* | GO:0034451 | CC | centriolar satellite | 0.040 | *PCM1* |
| GO:1901223 | BP | negative regulation of NIK/NF-kappaB signaling | 0.031 | *PPM1A* | GO:0050661 | MF | NADP binding | 0.004 | *CRYZL1/SPR* |
| GO:0007179 | BP | transforming growth factor beta receptor signaling pathway | 0.031 | *GIPC1/PPM1A* | GO:0004724 | MF | magnesium-dependent protein serine/threonine phosphatase activity | 0.020 | *PPM1A* |
| GO:0046599 | BP | regulation of centriole replication | 0.033 | *VPS4B* | GO:0048407 | MF | platelet-derived growth factor binding | 0.022 | *COL6A1* |
| GO:1990182 | BP | exosomal secretion | 0.033 | *VPS4B* | GO:0004033 | MF | aldo-keto reductase (NADP) activity | 0.033 | *SPR* |
| GO:2000810 | BP | regulation of bicellular tight junction assembly | 0.033 | *SNAI1* | GO:0070566 | MF | adenylyltransferase activity | 0.041 | *FICD* |
| GO:0021846 | BP | cell proliferation in forebrain | 0.034 | *PCM1* | GO:0000287 | MF | magnesium ion binding | 0.043 | *DCTPP1/PPM1A* |
| GO:0042438 | BP | melanin biosynthetic process | 0.034 | *GIPC1* | GO:0070412 | MF | R-SMAD binding | 0.043 | *PPM1A* |
| GO:0046827 | BP | positive regulation of protein export from nucleus | 0.034 | *PPM1A* |  |  |  |  |  |

Abbreviation: GOID, gene ontology id; BP, biological function; CC, cellular component; MF, molecular function.


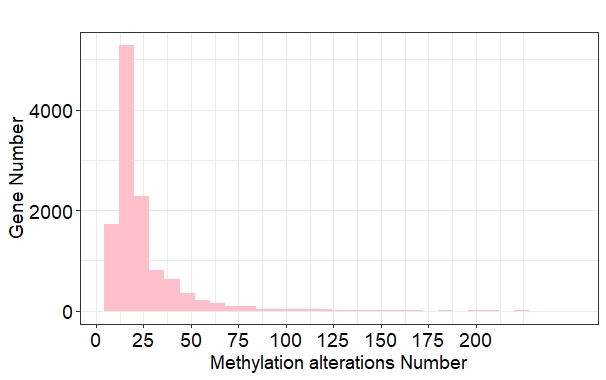


Figure S1. Number distribution of DNA methylation CpG sites within the promoter region and the gene body of the methylation-regulated gene.
